# The application of objective clinical human reliability analysis (OCHRA) in the assessment of basic robotic surgical skills

**DOI:** 10.1007/s00464-023-10510-2

**Published:** 2023-11-06

**Authors:** Jack Gorard, Matthew Boal, Vishaal Swamynathan, Walaa Ghamrawi, Nader Francis

**Affiliations:** 1 Division of Surgery & Interventional Science, Royal Free Hospital Campus, University College London, London, UK; 2 The Griffin Institute, Northwick Park and St Mark’s Hospital, London, UK; 3 Wellcome/EPSRC Centre for Interventional and Surgical Sciences, Charles Bell House, University College London, London, UK

**Keywords:** Error analysis, OCHRA, Robotic surgery, Assessment tools, Surgical training

## Abstract

**Background:**

Using a validated, objective, and standardised assessment tool to assess progression and competency is essential for basic robotic surgical training programmes. Objective clinical human reliability analysis (OCHRA) is an error-based assessment tool that provides in-depth analysis of individual technical errors. We conducted a feasibility study to assess the concurrent validity and reliability of OCHRA when applied to basic, generic robotic technical skills assessment.

**Methods:**

Selected basic robotic surgical skill tasks, in virtual reality (VR) and dry lab equivalent, were performed by novice robotic surgeons during an intensive 5-day robotic surgical skills course on da Vinci® X and Xi surgical systems. For each task, we described a hierarchical task analysis. Our developed robotic surgical-specific OCHRA methodology was applied to error events in recorded videos with a standardised definition. Statistical analysis to assess concurrent validity with existing tools and inter-rater reliability were performed.

**Results:**

OCHRA methodology was applied to 272 basic robotic surgical skills tasks performed by 20 novice robotic surgeons. Performance scores improved from the start of the course to the end using all three assessment tools; Global Evaluative Assessment of Robotic Skills (GEARS) [VR: *t*(19) = − 9.33, *p* < 0.001] [dry lab: *t*(19) = − 10.17, *p* < 0.001], OCHRA [VR: *t*(19) = 6.33, *p* < 0.001] [dry lab: *t*(19) = 10.69, *p* < 0.001] and automated VR [VR: *t*(19) = − 8.26, *p* < 0.001]. Correlation analysis, for OCHRA compared to GEARS and automated VR scores, shows a significant and strong inverse correlation in every VR and dry lab task; OCHRA vs GEARS [VR: mean *r* = − 0.78, *p* < 0.001] [dry lab: mean *r* = − 0.82, *p* < 0.001] and OCHRA vs automated VR [VR: mean *r* = − 0.77, *p* < 0.001]. There is very strong and significant inter-rater reliability between two independent reviewers (*r* = 0.926, *p* < 0.001).

**Conclusion:**

OCHRA methodology provides a detailed error analysis tool in basic robotic surgical skills with high reliability and concurrent validity with existing tools. OCHRA requires further evaluation in more advanced robotic surgical procedures.

**Supplementary Information:**

The online version contains supplementary material available at 10.1007/s00464-023-10510-2.

Cumulatively, over ten million robot-assisted procedures have been performed worldwide using da Vinci® surgical systems [1]. Surgeons have been quick to adopt robot-assisted surgery across various surgical specialities due to enhanced optics, greater freedom of movement and preferable ergonomics [2]. Despite the rapid uptake of robot-assisted procedures, training curriculums and consensus on assessments that can be implemented to inform practice and assist accreditation are still in their infancy. Training often varies vastly and can lack structure, with most surgeons undergoing mentorship and observation instead of specifically set performance targets [2]. There are accessible training courses; however, content again varies greatly, and the practical training offered is inconsistent [3]. In the United States, several adverse events in robot-assisted surgery were reported from 2000 to 2013 by Alemzadeh et al. using Food and Drug Administration data on the Manufacturer and User Facility Device Experience database [4]. A lack of structured training has partly contributed to this, and it was proposed that the implementation of uniform standards for training would reduce the incidence of adverse events [4]. Recently, the Orsi Academy and the British Association of Urological Surgeons have made vital progress in setting foundations for standardising robotic training curricula [5, 6].

Using a validated, objective and standardised assessment tool to assess progression and competency is essential for robotic surgical training programmes [7]. Objective clinical human reliability analysis (OCHRA) is an error-based hierarchical task analysis assessment tool that has been applied to laparoscopic surgery to detect and analyse technical errors with their consequences. This provides an in-depth analysis that is required for meaningful feedback. It is also based on granular observational data capture which provides detailed assessment of surgical performance.

OCHRA has demonstrated construct and predictive validity, with excellent intra- and inter-rater reliability in multiple studies across various surgical specialities for open and laparoscopic procedures [8–13]. It is more objective than global observation assessments as is it based on granular observational data capture which provides a detailed assessment of surgical performance. Automated performance metrics (APMs), namely kinematic and system event data from recording devices such as the dVLogger®, provide an exciting prospect for future training [14]. OCHRA can facilitate the application of artificial intelligence (AI) and machine learning to complement and advance the early efforts along with APMs, which have demonstrated construct and predictive validity in robotic surgery [15–17]. By embracing big data methodology such as deep learning, effective and meaningful large-scale performance feedback is on the horizon [18]. For AI to recognise and interpret errors, large, manually annotated dry lab and clinical videos are required to train and test models. OCHRA is most likely the only granular error analysis tool that has been validated within minimally invasive surgery to date, but no prior reports in robotic surgery.

The aims of this study were to develop an OCHRA methodology that is applicable to assess generic basic robot-assisted skills and to test its feasibility, concurrent validity and reliability for the assessment of technical performance in robot-assisted surgical tasks.

## Methodology

To explore the concurrent validity of OCHRA within basic robotic technical skills assessment, this study uses automated VR assessment scores and Global Evaluative Assessment of Robotic Skills (GEARS). VR scores have demonstrated face, content and predictive validity [19–23] and GEARS is one of the most widely used valid and reliable tools for robotic surgical assessments [24, 25].

### Development of OCHRA methodology

Our OCHRA methodology was adapted and refined from laparoscopic surgery, described by Tang, Miskovic and Foster [8, 13, 26], to be applied to robotic surgical procedures. Five categorisations for error events provide the OCHRA methodology framework: error type, consequence of error, external error mode, instrument and non-error event (Supplementary Table 1A–E). As this was a feasibility study, OCHRA experts improved and refined the bespoke robotic-specific methodology, which contained five domains of error events described in the laparoscopic OCHRA methodology [8, 13, 26]. This was done after initial analysis of the videos through author discussion and piloted within the robotic setting.

We describe error types for robotic surgical procedures as the observed error seen during video analysis. OCHRA error events contain a “consequence” domain, which would be designated a severity rating within clinical surgery [27]. However, this was deemed not feasible within basic robotic skills, as errors are usually binary with no true severity rating, therefore excluded from our analysis. External error modes are defined as the “external and observable manifestation of the error or behaviours exhibited by an operator i.e. the physical form an error takes” [28]. For laparoscopic surgery, Joice and Tang et al. describe ten generic forms of external error modes which represent “observed patterns of failure” and can be categorised into procedural or execution error modes relating to the observed “underlying causative mechanism” [10, 26].

### Task selection and hierarchical task analysis

**Fig. 1:**
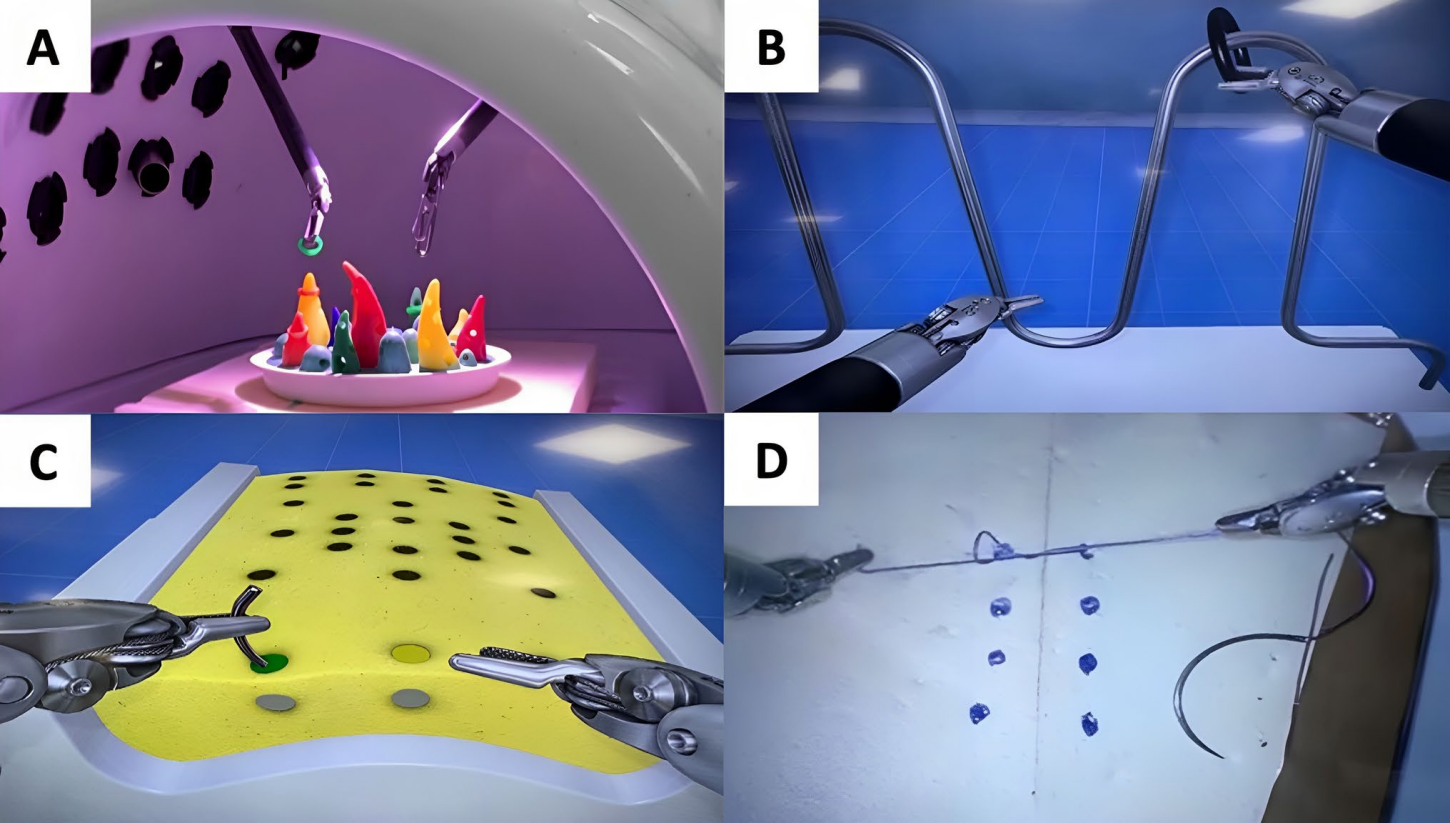
**A**, **D** are photographs of dry sea spikes and knot tying tasks being performed, respectively. **B**, **C** are photographs of VR ring rollercoaster tasks and big dipper needle driving tasks being performed, respectively

Eight robotic tasks in total were selected for this study which included four VR tasks and four dry lab equivalent tasks. They were sea spikes (Fig. 1A), ring rollercoaster (Fig. 1B), big dipper needle driving (Fig. 1C) and knot tying (Fig. 1D). These separate tasks necessitate a broad range of generic basic surgical skills for assessment; endowrist manipulation, simple grasping, instrument handling, camera control, clutching, needle control, needle driving and suture manipulation are all assessed to varying degrees.

For each, a hierarchical task analysis was created through expert consensus with two surgical specialists (Supplementary Table 2A–D). A validated and reliable robotic surgical suturing checklist provided the basic framework for developing our task analyses for big dipper needle driving and knot tying tasks [29]. To our knowledge, there are no validated checklists for sea spikes or ring rollercoaster tasks within the literature. Each task was divided into four sequential component task zones. Task zones were subdivided further into subtasks with the aim of each subtask clearly described.

### Study design and data collection

Participants were invited as part of a University Robotic Curriculum and a surgical specialty society robotic training course (The Association of Laparoscopic Surgeons of Great Britain and Ireland), with standardised timetable. An Institutional Review Board was not required as the study did not involve patient care. Ethics followed Helsinki principle, collecting informed written consent. Using definitions by Sridhar et al., we define novice robotic surgeons as those who have performed no robotic surgical procedures [30].

The 5-day robot-assisted surgical skills courses were held at a pre-clinical surgical research and training hub. All recorded and assessed VR tasks for this study were performed using da Vinci SimNow®. All recorded and assessed dry model tasks were performed using either da Vinci® X or Xi robotic surgical systems. VR and dry lab tasks were video recorded and uploaded by participants to a secure cloud database server.

Attempt 1, for both VR and dry tasks, occurred after orientation and training on the 1st day of the course with Attempt 2 on the 5th day. Each was recorded and assessed using GEARS for dry lab and VR tasks, and automated VR scores. GEARS assessments were carried out by two trained, independent, surgical residents. GEARS scores were obtained using a five-point Likert scale indicating competency in six domains: depth perception, bimanual dexterity, efficiency, force sensitivity, autonomy and robotic control, with a maximum score of thirty. Da Vinci SimNow® provided automated VR scores incorporating the total time to complete the task (scored out of fifty) and economy of motion (scored out of fifty), with a maximum score of one hundred. OCHRA error scores were obtained from analysing video recordings retrospectively after the course. The OCHRA assessor was blinded to other assessment scores.

### OCHRA specialist training

Two independent assessors underwent specialist OCHRA training by specialists. Inter-rater reliability was tested and reviewed to improve assessment before reviewing all videos. OCHRA was applied to videos of each task by the two assessors independently. Eleven video tasks were compared using OCHRA methodology by the independent assessors with an 80.9% percentage agreement for matched error events [182/216 (86.1%) error events matched].

### Statistical analysis

Paired *T* test was used to assess each participant’s change in the sum of scores for each assessment tool from Attempt 1 to Attempt 2 at two separate points along the participant’s learning curve. Wilcoxon matched-pair signed-rank test, the non-parametric and thus assumption-free counterpart of paired t tests, was conducted to validate findings. Using multiple imputation (regression method), missing data sets were accounted for when using Paired *T* test, Wilcoxon matched-pair signed-rank test and generating graphs.

For each selected task, correlational analysis examined the degree to which scores from each separate assessment tool correlated. Spearman correlation coefficients were consistent with the direction and magnitude of Pearson correlations, but Spearman correlation coefficients were reported instead of Pearson’s given the sample size. Inter-rater reliability was assessed using Spearman correlation coefficient. Two-tailed *p* values < 0.05 were considered significant.

All statistical analyses were carried out with SPSS Statistics 22.0 software (IBM corporation on and other(s) 1989, USA, 2013).

## Results

Twenty participants age ranged from 25 to 53 years old (mean 32) performed 320 robotic skills tasks. There was an even split of female to male participants with 10 (50%) females. Participants were all qualified doctors and undertook their medical training in several different countries. The United Kingdom was the most popular with 10 (50%) doctors completing medical training there. Participants had varying open & laparoscopic surgical experience, and the majority, 12/20 (60%), had not completed any years of open or laparoscopic surgical experience. Only one participant had over 10 years of open & laparoscopic surgical experience. Importantly no participants had performed any robotic surgeries, so were all novice robotic surgeons.

### Collected data

320 selected robotic skills tasks were assessed, including 160 videos for VR and 160 videos for dry lab. OCHRA video analysis was applied to an estimated 1632 min (over 27 h) of video data with a total of 6488 error events analysed. GEARS scores were obtained for all 320/320 tasks (100%). A total of 48/320 (15%) videos were incomplete or missing for dry lab and VR tasks. Therefore, OCHRA video analysis was applied to 272/320 (85%) dry lab and VR tasks with OCHRA error scores recorded and automated VR scores were obtained for 148/160 (92.5%) VR tasks.

### Distribution of OCHRA error events

Error events most commonly occurred in the following task zones: for sea spikes [VR: ‘(2) Movement of ring in space’, 245/677 error events (36.2%)] [Dry lab: ‘(2) Movement of ring in space’, 186/582 error events (32.0%)], for ring rollercoaster [VR: ‘(2) Movement of ring along rollercoaster’, 805/1004 error events (80.2%)] [Dry lab: ‘(2) Movement of ring along rollercoaster’, 702/900 error events (78.0%)], for big dipper needle driving [VR: ‘(4) Exit of needle from tissue’, 813/1771 error events (45.9%)] [Dry lab: ‘(1) Preparing needle for tissue insertion’, 368/939 error events (39.2%)] and for knot tying [VR: ‘(2) Wrapping suture thread around the instrument’, 243/647 error events (37.6%)] [Dry lab: ‘(1) Preparing suture thread for making loops’, 126/324 error events (38.9%)].

The most common error type was ‘(*N*) Inappropriate/poor use of endowristed instrument’ for each task in both VR and dry lab. Specifically, for sea spikes [VR: 246/677 error events (36.3%)] [Dry lab: 258/582 error events (44.3%)], for ring rollercoaster [VR: 887/1004 error events (88.3%)] [Dry lab: 823/900 error events (91.4%)], for big dipper needle driving [VR: 633/1771 error events (35.7%)] [Dry lab: 532/939 error events (56.7%)] and for knot tying [VR: 292/647 error events (45.1%)] [Dry lab: 152/324 error events (46.9%)].

The most common consequence for sea spikes was ‘(15) Delay in progress of procedure’ in both VR and dry lab [VR: 368/677 error events (54.4%)] [Dry lab: 404/582 error events (69.4%)], for ring rollercoaster was ‘(13) Risk of injury to other structure/minor collision’ in both VR and dry lab [VR: 838/1004 error events (83.5%)] [Dry lab: 803/900 error events (89.2%)], for big dipper needle driving was ‘(13) Risk of injury to other structure/minor collision’ in VR [VR: 820/1771 error events (46.3%)] and ‘(8) Tissue/foam pad damaged’ in dry lab [Dry lab: 360/939 error events (38.3%)] and for knot tying was ‘(15) Delay in progress of procedure’ in both VR and dry lab [VR: 329/647 error events (50.9%)] [Dry lab: 187/324 error events (57.7%)].

External error modes can be divided into procedural errors (a–f) and executional errors (g–j). The executional errors (g–j) were greater than procedural errors (a–f) in each task; sea spikes executional errors [VR: 418/677 error events (61.7%)] [Dry lab: 335/582 error events (57.6%)], ring rollercoaster executional errors [VR: 925/1004 error events (92.1%)] [Dry lab: 860/900 error events (95.6%)], big dipper needle driving executional errors [VR: 1572/1771 error events (88.8%)] [Dry lab: 786/939 error events (83.7%)] and knot tying executional error events [VR: 496/647 error events (76.7%)] [Dry lab: 233/324 error events (71.9%).

The only instruments used for each task were the ‘(6) Fine grasper (Marylands, needle driver)’ and ‘(15) Camera endoscope’. Instruments were not exchanged during any of the tasks. A total 6373/6844 error events (93.1%) were caused using ‘(6) Fine grasper (Marylands, needle driver)’ across all tasks.

The most common non-error event for sea spikes was ‘(f) Continue uninterrupted’ in both VR and dry lab [VR: 347/677 error events (51.3%)] [Dry lab: 244/582 error events (41.9%)], for ring rollercoaster was ‘(b) Adjust hold to improve orientation’ in both VR and dry lab [VR: 798/1004 error events (79.5%)] [Dry lab: 762/900 error events (84.7%)], for big dipper needle driving ‘(f) Continue uninterrupted’ in VR [VR: 725/1771 error events (40.9%)] and ‘(j) Corrective action within subtask’ in dry lab [Dry lab: 329/939 error events (35.0%)], and for knot tying ‘(h) Requires repetition of step’ in both VR and dry lab [VR: 237/647 error events (36.6%)] [Dry lab: 111/324 error events (34.3%)].

### Learning curve of participants

**Fig. 2:**
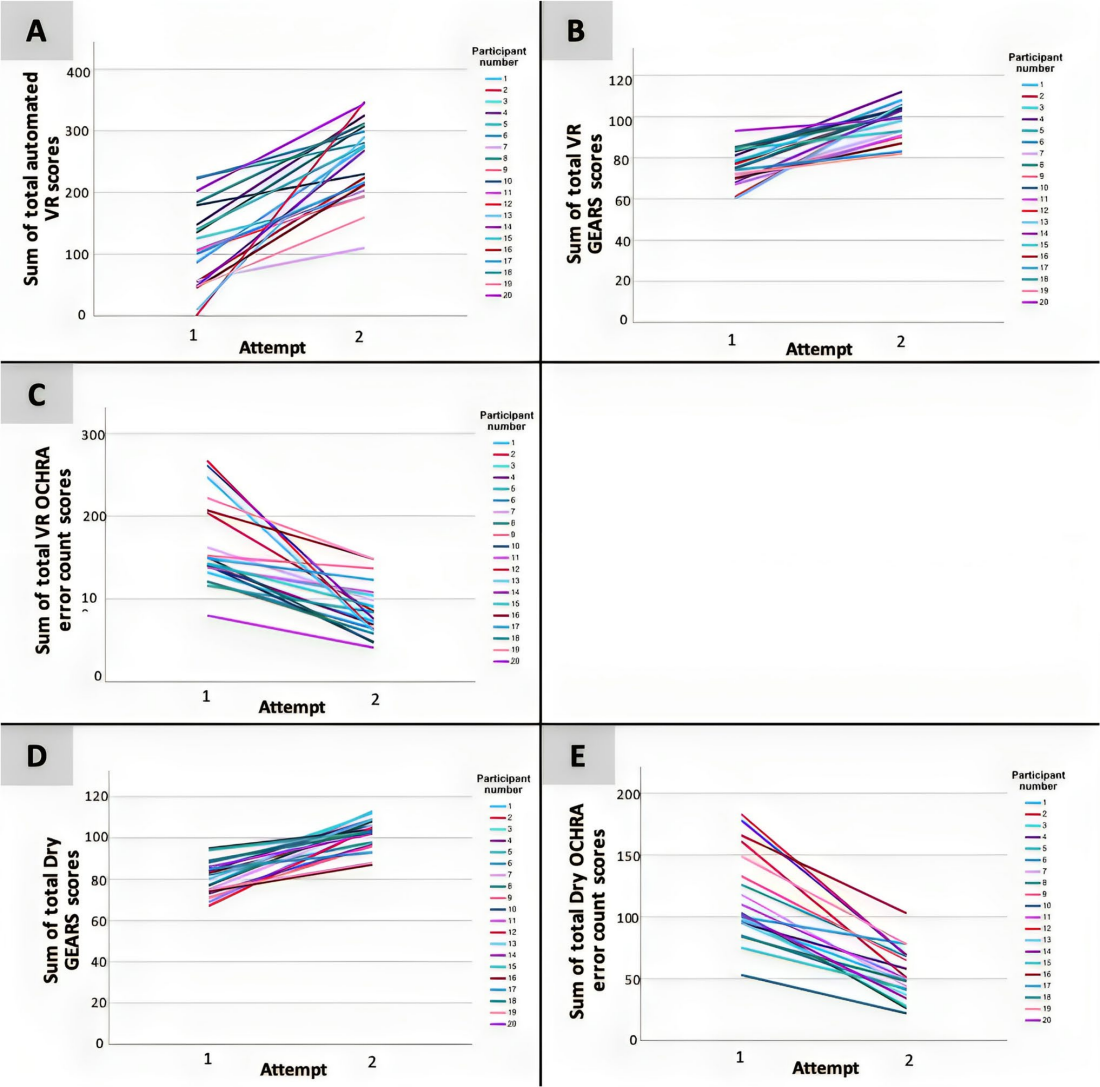
Performance change from start of the course (Attempt 1) to end of course (Attempt 2) using the different assessment tools for each participant. **A**–**C** Show performance change for VR tasks using each assessment tool; sum of automated VR scores, sum of GEARS scores and sum of OCHRA scores, respectively, for each participant. **D**, **E** Show performance change for dry lab tasks using each assessment; sum of GEARS scores and sum of OCHRA scores, respectively, for each participant

Performance improved from the start of the course (Attempt 1) and the end of the week (Attempt 2) using all assessment tools for each candidate (Fig. 2A–E). Paired samples t tests indicated that participants significantly improved (all two-tailed *p* values < 0.001) over the course using each assessment tool in both VR and dry lab tasks (Table 1). The mean difference ± standard deviation for the difference from the sum of attempt 1 assessment scores to the sum of attempt 2 assessment scores was 78.50 ± 55.49 for VR OCHRA error count scores, − 23.18 ± 11.11 for VR GEARS scores, − 142.57 ± 77.15 for automated VR scores, 62.70 ± 26.22 for dry lab OCHRA error count scores and − 21.95 ± 9.65 for dry lab GEARS scores. The median difference (interquartile range) for the difference from the sum of attempt 1 assessment scores to the sum of attempt 2 assessment scores was 61 (64.50) for VR OCHRA error count scores, − 22 (14.75) for VR GEARS scores, − 132.5 (96.38) for automated VR scores, 61.5 (34.75) for VR OCHRA error count scores and − 22 (17.25) for dry lab GEARS scores. Wilcoxon matched-pairs signed-rank tests, the non-parametric and thus assumption-free counterpart of paired t tests validated these findings (Table 2).

**Table 1:** Paired sample *T* test comparing sum of participant’s assessment scores from Attempt 1 to Attempt 2 for each assessment tool

	*N*	Mean diff.	Std. deviation	Median diff.	Inter-quartile range	Std. error mean	95% confidence interval of the difference		*t*	*df*	Sig. (2-tailed) *p* value
							Lower	Upper			
Sum of Attempt 1 automated VR scores	20	− 142.57	77.15	− 132.50	96.38	17.25	− 178.68	− 106.46	− 8.26	19	< 0.001
Sum of Attempt 2 automated VR scores	20										
Sum of Attempt 1 VR GEARS scores	20	− 23.18	11.11	− 22.00	14.75	2.48	− 28.27	− 17.98	− 9.33	19	< 0.001
Sum of Attempt 2 VR GEARS scores	20										
Sum of Attempt 1 VR OCHRA scores	20	78.50	55.49	61.00	64.50	12.41	52.53	104.47	6.33	19	< 0.001
Sum of Attempt 2 VR OCHRA scores	20										
Sum of Attempt 1 dry GEARS scores	20	− 21.95	9.65	− 22.00	17.25	2.16	− 26.47	− 17.43	− 10.17	19	< 0.001
Sum of Attempt 2 dry GEARS scores	20										
Sum of Attempt 1 dry OCHRA scores	20	62.70	26.22	61.50	34.75	5.86	50.43	74.97	10.69	19	< 0.001
Sum of Attempt 2 dry OCHRA scores	20

**Table 2:** Related-samples Wilcoxon signed rank test summary, the non-parametric, comparing sum of assessment scores for Attempt 1 and 2 for each assessment tool in VR and dry lab

	Sum of Attempt 1 automated VR scores & sum of Attempt 2 automated VR scores	Sum of Attempt 1 VR GEARS scores & sum of Attempt 2 VR GEARS scores	Sum of Attempt 1 VR OCHRA error count scores & sum of Attempt 2 VR OCHRA error count scores	Sum of Attempt 1 dry GEARS scores & sum Attempt 2 dry GEARS scores	Sum of Attempt 1 dry OCHRA error count scores & sum of Attempt 2 dry OCHRA error count scores
Total *N*	20	20	20	20	20
Test statistic	210.00	210.00	0.00	210.00	0.00
Standard error	26.79	26.78	26.78	26.77	26.78
Standardised test statistic	3.92	3.92	− 3.92	3.92	− 3.92
Asymptotic sig. (2-sided test)	< 0.001	< 0.001	< 0.001	< 0.001	< 0.001

Related-samples Wilcoxon signed rank test summary, the non-parametric, is used to validate the paired *T* test findings. The null hypothesis (the median of difference between sum of Attempt 1 scores and sum Attempt 2 scores equal 0) can be rejected with significance < 0.001 for each assessment tool in both VR and dry lab

### Concurrent validity of OCHRA

**Fig. 3:**
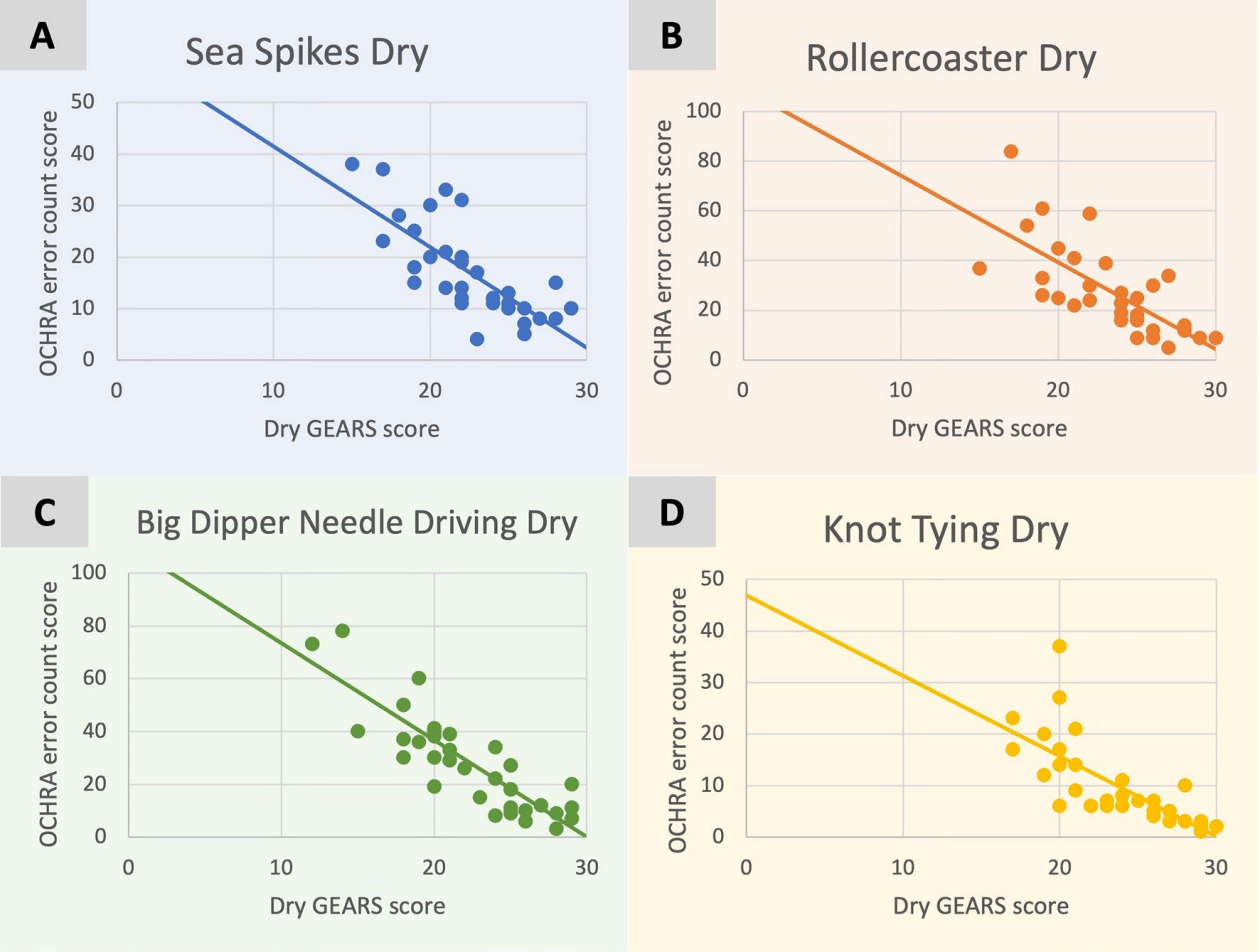
OCHRA error count scores compared to dry GEARS scores for sea spikes dry tasks (**A**), ring rollercoaster dry tasks (**B**), big dipper needle driving dry tasks (**C**) and knot tying dry tasks (**D**). Each point represents a different participant. The OCHRA error count maximum score is infinite. The dry GEARS maximum score is 30

Strong inverse and significant associations were seen between dry OCHRA error count scores and dry GEARS scores for dry lab tasks (range *r* = − 0.86 to − 0.76, mean *r* = − 0.82) with a breakdown seen in Table 3 and Fig. 3A–D.

**Table 3:** Correlation analysis using Spearman’s rho correlation coefficient to examine concurrent validity in scores between assessment tools for the different tasks in both VR and dry lab

	Dry OCHRA error count scores compared to dry GEARS scores			VR OCHRA error count scores compared to VR GEARS scores			VR OCHRA error count scores compared automated VR scores			VR GEARS scores compared to automated VR scores		
	*N*	*r*Spearman	*p* value	*N*	*r*Spearman	*p* value	*N*	*r*Spearman	*p* value	*N*	*r*Spearman	*p* value
Sea spikes	35	− 0.81	< 0.001	38	− 0.67	< 0.001	37	− 0.85	< 0.001	38	0.71	< 0.001
Ring rollercoaster	31	− 0.76	< 0.001	35	− 0.84	< 0.001	33	− 0.64	< 0.001	36	0.68	< 0.001
Big dipper needle driving	34	− 0.85	< 0.001	29	− 0.92	< 0.001	28	− 0.90	< 0.001	35	0.88	< 0.001
Knot tying	33	− 0.86	< 0.001	36	− 0.70	< 0.001	36	− 0.67	< 0.001	39	0.82	< 0.001
Mean		− 0.82			− 0.78			− 0.77			0.77

**Fig. 4:**
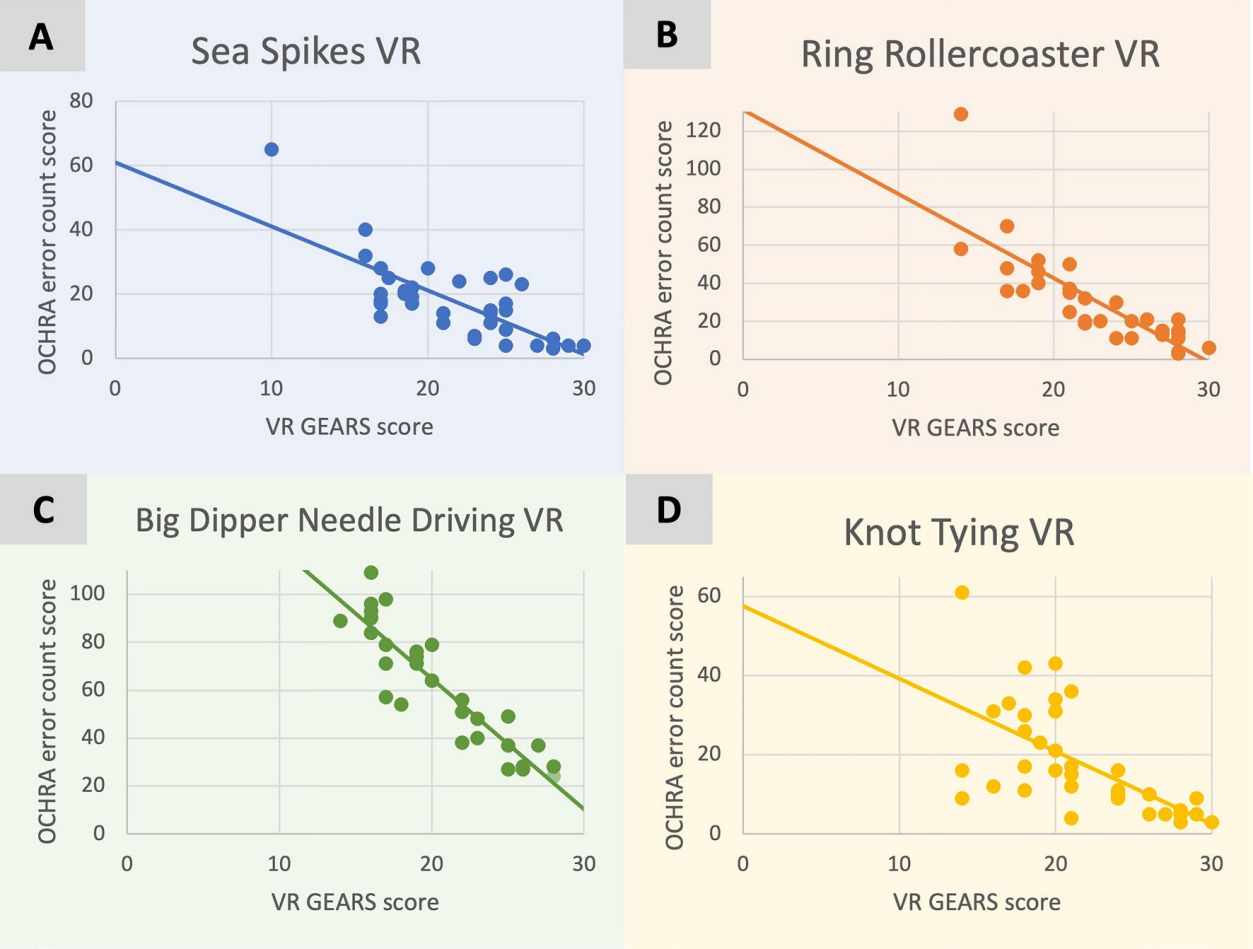
OCHRA error count scores compared to VR GEARS scores for sea spikes VR tasks (**A**), ring rollercoaster VR tasks (**B**), big dipper needle driving VR tasks (**C**) and knot tying VR tasks (**D**). Each point represents a different participant. The OCHRA error count maximum score is infinite. The VR GEARS maximum score is 30

Strong inverse and significant associations were seen between VR OCHRA error count scores and VR GEARS scores for VR tasks (range *r* = − 0.92 to − 0.67, mean *r* = − 0.78) with a breakdown seen in Table 3 and Fig. 4A–D.

**Fig. 5:**
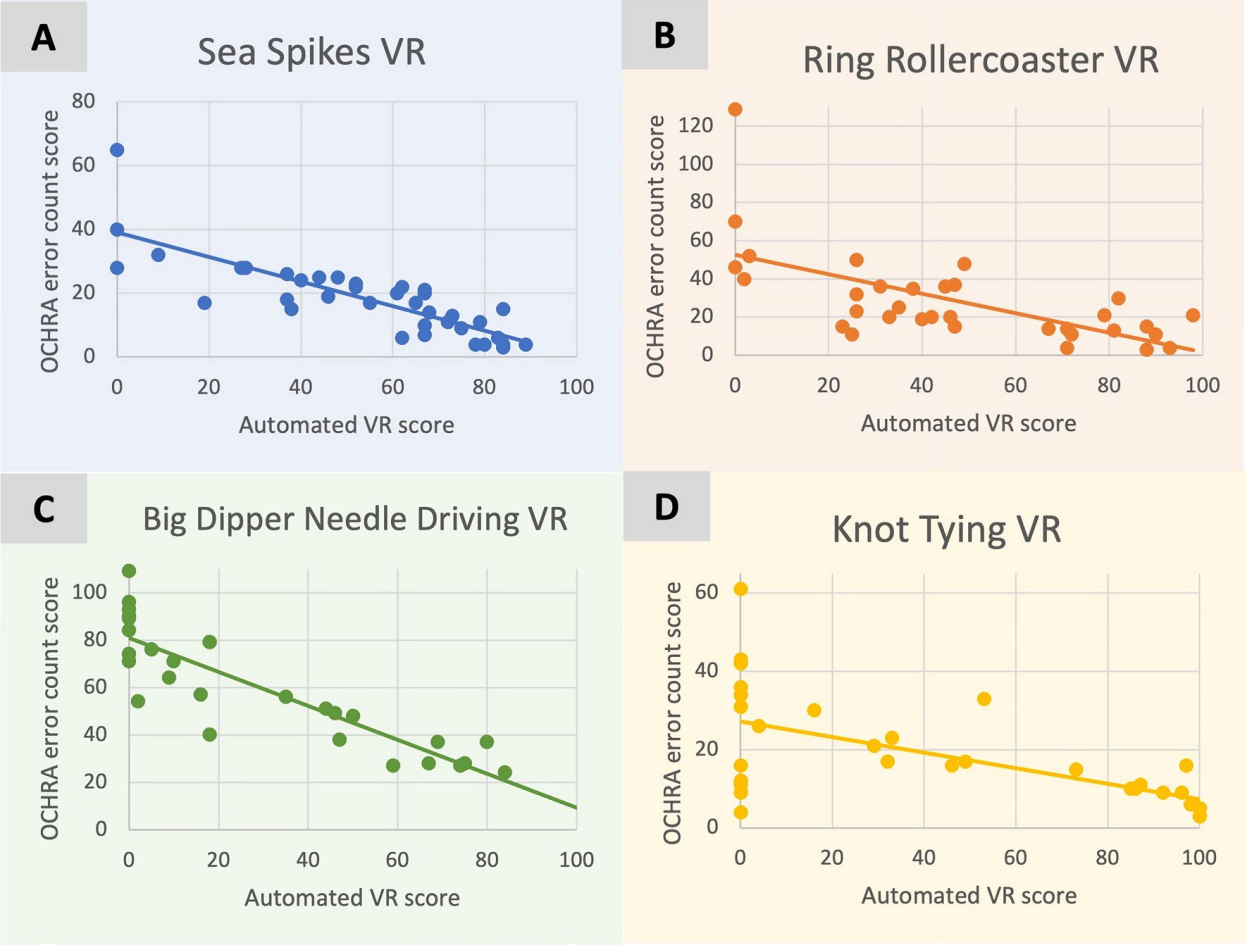
OCHRA error count scores compared to automated VR scores for sea spikes VR tasks (**A**), ring rollercoaster VR tasks (**B**), big dipper needle driving VR tasks (**C**) and knot tying VR tasks (**D**). Each point represents a different participant. The OCHRA error count maximum score is infinite. The automated VR maximum score is 100

Strong inverse and significant associations were seen between VR OCHRA error count scores and automated VR scores for VR tasks (range *r* = − 0.90 to − 0.64, mean *r* = − 0.77) with a breakdown seen in Table 3 and Fig. 5A–D.

**Fig. 6:**
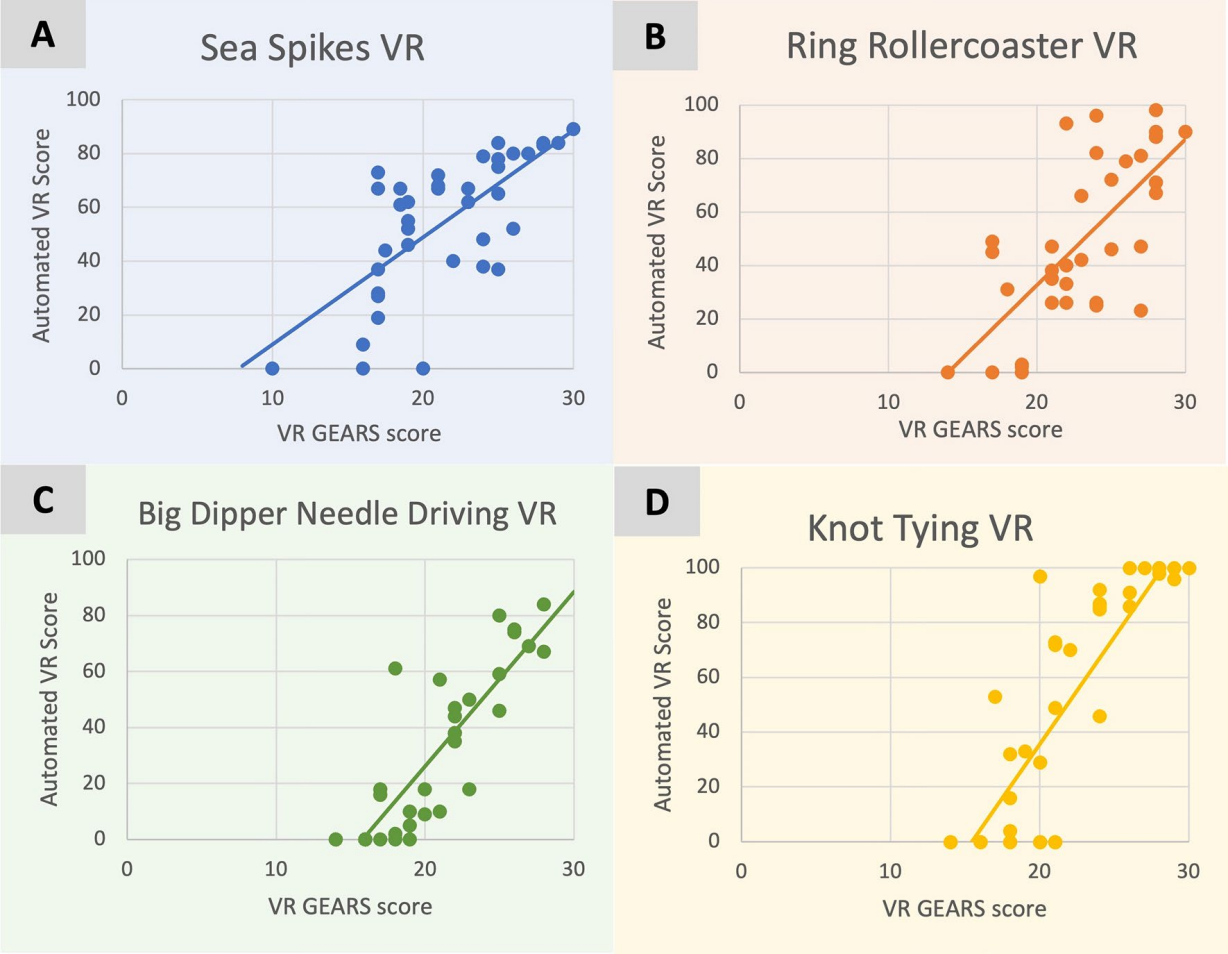
Automated VR scores compared to VR GEARS scores for sea spikes VR tasks (**A**), ring rollercoaster VR tasks (**B**), big dipper needle driving VR tasks (**C**) and knot tying VR tasks (**D**). Each point represents a different participant. The automated VR maximum score is 100. The VR GEARS maximum score is 30

Strong and significant associations were seen between VR GEARS error count scores and automated VR scores for VR task (range *r* = 0.68 to 0.88, mean *r* = 0.77) with a breakdown seen in Table 3 and Fig. 6A–D.

### Inter-rater reliability

There is a very strong positive correlation between reviewer 1 and reviewer 2 (*r*Spearman (11) = 0.926, *p* < 0.001) such that both reviewers scored participants similarly showing very strong inter-rater reliability (Table 4).

**Table 4:** Correlation analysis using Spearman’s rho correlation coefficient to examine the inter-rater reliability between two independent assessors for OCHRA error scores

	Spearman’s rho correlation coefficient	Significance (2-tailed)	95% confidence intervals (2-tailed)	
			Lower	Upper
OCHRA reviewer 1 *vs* OCHRA reviewer 2 (*N* = 11)	0.926	< 0.001	0.724	0.982

At least one of each selected task in VR and dry lab equivalent were analysed by both reviewers independently

## Discussion

This feasibility study is the first to investigate the application of OCHRA as an assessment tool in robotic surgery. Our developed bespoke robotic OCHRA methodology has shown concurrent validity with GEARS and automated VR scores, plus excellent inter-rater reliability. Furthermore, it has shown the ability to demonstrate participants’ proficiency learning curve. This OCHRA tool has the potential to be applied within the rapidly expanding domain of AI as a valuable educational adjunct to inform deep learning and robotic surgery accreditation programmes.

Using a validated, objective and standardised assessment tool to assess progression and competency is essential for robotic surgical training programmes [7]. Global rating scales lack the in-depth granular analysis of technical skill and objectivity that error-based analysis provides [31]. Despite the plethora of publications on assessment tools in robotic surgery [18, 25, 32–39], there is a lack of error-based analysis methods in robotic surgery for education or accreditation. For error assessment methods to be utilised widely, a clear definition of what is considered an error is required. In addition to this, an error tool needs to be generic and adaptable to multiple procedures whilst retaining its objectivity.

Current technical error-based assessment tools for minimally invasive surgery include Generic Error Rating Tool and Fundamentals of Laparoscopic Surgery. They provide basic error analysis by identifying that an observed error event has occurred and in what step of the procedure [31, 40, 41]. OCHRA methodology analyses events further by analysing the consequences of the error, the severity of the error, the mechanism by the operator which resulted in the error, the instrument which caused the error and the corrective events following the error. Assessing each error in such detail aids in more meaningful feedback which is fundamental to learning and for future application of AI methods. Our analyses showed executional errors were far more common than procedural errors within external error modes. Differentiating between procedural and executional errors is valuable for identifying areas to improve and reduce errors [26]. Executional errors suggest a lack of technical skills or inadequate equipment, whereas procedural errors suggest a lack of knowledge understanding the steps of a procedure.

Due to this granularity, OCHRA is likely to complement the assessment that is most commonly used in robotic surgery, the GEARS tool. It has been postulated that the ability to identify errors is linked to surgical aptitude [42, 43] with recent research using OCHRA proving a direct link between intra-operative performance and errors to patient outcomes, demonstrating predictive validity [12, 17, 44]. Incorporating error-based assessments such as OCHRA into robotic surgical training may accelerate the proficiency learning curves for trainees, as in-depth analysis of individual mistakes will enable surgeons to learn from and prevent them. Consequently, this can underpin future research that aims to improve patient safety in the operating room and within robotic surgical training [45].

Preventing a higher uptake of OCHRA within surgical training programmes is the substantial time required to analyse surgical videos. Additionally, OCHRA requires prior training on the methodology and its application to manually annotate and code the errors including the grading, the severity and any consequences. Moreover, OCHRA assessments cannot provide immediate feedback like GEARS and automated VR scores can, hence, precluding its use as a formative tool. Therefore, it may be better suited for objective, summative competency assessments that can be used for accreditation in specific procedures. The National Training Programme for laparoscopic colorectal surgery (LAPCO programme) is an example of an effective training programme which uses summative competency assessments for accreditation [46, 47]. The next stage of evaluation of OCHRA in robotic surgery is to demonstrate its construct validity with clinically relevant metrics.

The application of OCHRA to inform AI models is an exciting prospect. OCHRA can provide granularly annotated surgical videos, particularly in this context, to recognise a near miss or a consequential error. The long-term aim is to have an early warning system with real-time operative feedback. This is a long way off, however, OCHRA potentially can provide an opportunity to train deep learning methods in the recognition of errors and near misses. Further large studies and fully validated data sets of objectively analysed operative videos are required to allow this progression, to fully automated, objective, meaningful performance assessments.

This study had certain limitations, despite a large number of videos analysed, over 27 h, using OCHRA methodology, 20 participants performed the selected tasks in this proof-of-concept study. Research with larger sample sizes and different levels of experience is required before application in clinical practice and training programmes for robotic surgery.

Although this study confirmed the feasibility of recording the tasks, collating and analysing the data, there were 48 videos out of 320 (15%) of the selected tasks were not able to undergo OCHRA analysis due to missing data or incomplete videos. This was mainly because of the use of external recording, but in future studies, recording will use direct video capture.

This study may also lack external validity, as it used novice robotic surgical trainees as participants. In addition to this, we have not established construct or predictive validity for OCHRA in robotic surgical skill analysis, although presumably has a high likelihood of doing so, as it did in laparoscopic surgery [11–13].

The selected tasks used required a suitable range of general and basic robotic surgical skills. However, more advanced skills such as diathermy and dissection were not assessed in any of the selected tasks. Further work is needed to investigate if OCHRA methodology is applicable to more complex tasks that require more advanced robotic skills. This was a feasibility study which was intended to test the application of the OCHRA methodology outside clinical practice in the first instance. Since demonstrating the feasibility, reliability and validity of the OCHRA methodology in the lab setting, we are now proceeding with its application in a multi-centre clinical study, which is currently recruiting (Video Analysis in Minimally Invasive Surgery) (IRAS number 309024 and ClinicalTrials.gov Identifier: NCT05279287).

Whilst we have demonstrated excellent inter-rater reliability for evaluating error frequencies, there is potential for variability if other observers’ scores use a different definition of what constitutes an error. In our task analyses, we clearly describe the correct action to complete each subtask to minimise inter-rater variability using the OCHRA methodology. It is essential when using OCHRA methodology for other robotic surgical tasks that these descriptions are clear and well-defined within task analysis subtasks.

## Conclusion

This study shows the feasibility, concurrent validity and excellent reliability of OCHRA methodology to assess technical performance of basic robotic surgical skills. Further application of OCHRA to more advanced robotic surgical procedures is required to validate its future application in the operating room, and the application of AI to evaluate automated error recognition.

### Supplementary Information

Below is the link to the electronic supplementary material.

Supplementary file1 (DOCX 26 kb)

Supplementary file2 (DOCX 31 kb)

## Abbreviations

AI: Artificial intelligence
APMs: Automated performance metrics
BAUS: British Association of Urological Surgeons
FDA: Food and Drug Administration
FLS: Fundamentals of Laparoscopic Surgery
GEARS: Global Evaluative Assessment of Robotic Skills
GERT: Generic Error Rating Tool
IRB: Institutional Review Board
LAPCO: The National Training Programme for laparoscopic colorectal surgery
MAUDE: Manufacturer and User Facility Device Experience
OCHRA: Objective clinical human reliability analysis
VR: Virtual reality

## References

[1] Intuitive Surgical Inc (2021) Intuitive reaches 10 million procedures performed using da Vinci Surgical Systems. https://isrg.intuitive.com/news-releases/news-release-details/intuitive-reaches-10-million-procedures-performed-using-da-vinci

[2] Dixon F, Keeler BD (2020). Robotic surgery: training, competence assessment and credentialing. Bull R Coll Surg Engl.

[3] Chen R, Rodrigues Armijo P, Krause C, Siu KC, Oleynikov D (2020). A comprehensive review of robotic surgery curriculum and training for residents, fellows, and postgraduate surgical education. Surg Endosc.

[4] Alemzadeh H, Raman J, Leveson N, Kalbarczyk Z, Iyer RK (2016). Adverse events in robotic surgery: a retrospective study of 14 years of FDA data. PLoS ONE.

[5] Vanlander AE, Mazzone E, Collins JW, Mottrie AM, Rogiers XM, van der Poel HG, Van Herzeele I, Satava RM, Gallagher AG (2020). Orsi Consensus Meeting on European Robotic Training (OCERT): results from the first multispecialty consensus meeting on training in robot-assisted surgery. Eur Urol.

[6] Challacombe B, Ahmed K, Soomro N, Dasgupta P, Shamim Khan M, Cross W, Weston R, Sanger V, Joyce A, O’Flynn K, Speakman M (2015) British Association of Urological Surgeons (BAUS) Robotic surgery curriculum—guidelines for training. https://www.baus.org.uk/professionals/baus_business/publications/83/robotic_surgery_curriculum/

[7] Puliatti S, Mazzone E, Dell’Oglio P (2020). Training in robot-assisted surgery. Curr Opin Urol.

[8] Foster JD, Miskovic D, Allison AS, Conti JA, Ockrim J, Cooper EJ, Hanna GB, Francis NK (2016). Application of objective clinical human reliability analysis (OCHRA) in assessment of technical performance in laparoscopic rectal cancer surgery. Tech Coloproctol.

[9] Tang B, Hanna GB, Cuschieri A (2005). Analysis of errors enacted by surgical trainees during skills training courses. Surgery.

[10] Joice P, Hanna GB, Cuschieri A (1998). Errors enacted during endoscopic surgery—a human reliability analysis. Appl Ergon.

[11] Gauba V, Tsangaris P, Tossounis C, Mitra A, McLean C, Saleh GM (2008). Human reliability analysis of cataract surgery. Arch Ophthalmol.

[12] Curtis NJ, Dennison G, Brown CSB, Hewett PJ, Hanna GB, Stevenson ARL, Francis NK (2021). Clinical evaluation of intraoperative near misses in laparoscopic rectal cancer surgery. Ann Surg.

[13] Miskovic D, Ni M, Wyles SM, Parvaiz A, Hanna GB (2012). Observational clinical human reliability analysis (OCHRA) for competency assessment in laparoscopic colorectal surgery at the specialist level. Surg Endosc.

[14] Lyman WB, Passeri MJ, Murphy K, Siddiqui IA, Khan AS, Iannitti DA, Martinie JB, Baker EH, Vrochides D (2021). An objective approach to evaluate novice robotic surgeons using a combination of kinematics and stepwise cumulative sum (CUSUM) analyses. Surg Endosc.

[15] Hung AJ, Chen J, Gill IS (2018). Automated performance metrics and machine learning algorithms tomeasure surgeon performance and anticipate clinical outcomes in robotic surgery. JAMA Surg.

[16] Hung AJ, Liu Y, Anandkumar A (2021). Deep learning to automate technical skills assessment in robotic surgery. JAMA Surg.

[17] Hung AJ, Ma R, Cen S, Nguyen JH, Lei X, Wagner C (2021). Surgeon automated performance metrics as predictors of early urinary continence recovery after robotic radical prostatectomy—a prospective bi-institutional study. Eur Urol Open Sci.

[18] Chen J, Cheng N, Cacciamani G, Oh P, Lin-Brande M, Remulla D, Gill IS, Hung AJ (2019). Objective assessment of robotic surgical technical skill: a systematic review. J Urol.

[19] Hertz AM, George EI, Vaccaro CM, Brand TC (2018). Head-to-head comparison of three virtual-reality robotic surgery simulators. J Soc Laparosc Robot Surg.

[20] Abboudi H, Khan MS, Aboumarzouk O, Guru KA, Challacombe B, Dasgupta P, Ahmed K (2013). Current status of validation for robotic surgery simulators—a systematic review. BJU Int.

[21] Schmidt MW, Köppinger KF, Fan C, Kowalewski K-F, Schmidt LP, Vey J, Proctor T, Probst P, Bintintan VV, Müller-Stich B-P, Nickel F (2021). Virtual reality simulation in robot-assisted surgery: meta-analysis of skill transfer and predictability of skill. BJS Open.

[22] Guerin S, Huaulmé A, Lavoue V, Jannin P, Timoh KN (2022). Review of automated performance metrics to assess surgical technical skills in robot-assisted laparoscopy. Surg Endosc.

[23] Alshuaibi M, Perrenot C, Hubert J, Perez M (2020). Concurrent, face, content, and construct validity of the RobotiX Mentor simulator for robotic basic skills. Int J Med Robot Comput Assist Surg.

[24] Sánchez R, Rodríguez O, Rosciano J, Vegas L, Bond V, Rojas A, Sanchez-Ismayel A (2016). Robotic surgery training: construct validity of Global Evaluative Assessment of Robotic Skills (GEARS). J Robot Surg.

[25] Goh AC, Goldfarb DW, Sander JC, Miles BJ, Dunkin BJ (2012). Global evaluative assessment of robotic skills: validation of a clinical assessment tool to measure robotic surgical skills. J Urol.

[26] Tang B, Hanna GB, Joice P, Cuschieri A (2004). Identification and categorization of technical errors by observational clinical human reliability assessment (OCHRA) during laparoscopic cholecystectomy. Arch Surg.

[27] Francis NK, Curtis NJ, Conti JA, Foster JD, Bonjer HJ, Hanna GB, Abu-Hilal M, Agresta F, Antoniu SA, Arezzo A, Balagúe C, Boni L, Bouvy N, Carus T, Edwin B, Diana M, Faria G, Ignjatovic D, de Manzini N, Margallo FM, Martinek L, Matveev N, Mintz Y, Nakajima K, Popa DE, Schijven PJ, Sedman P, Yiannakopoulou E (2018). EAES classification of intraoperative adverse events in laparoscopic surgery. Surg Endosc.

[28] Salmon P, Stanton NA, Walker G (2003) Human factors design methods review. Human Factors Integration Defence Technology Centre. Ref HFIDTC/WPI.3./1. https://eprints.soton.ac.uk/368316/1/__soton.ac.uk_ude_personalfiles_users_jr1d11_mydesktop_ePrints_hf-design-methods-review.pdf

[29] Guni A, Raison N, Challacombe B, Khan S, Dasgupta P, Ahmed K (2018). Development of a technical checklist for the assessment of suturing in robotic surgery. Surg Endosc.

[30] Sridhar AN, Briggs TP, Kelly JD, Nathan S (2017). Training in robotic surgery—an overview. Curr Urol Rep.

[31] Husslein H, Shirreff L, Shore EM, Lefebvre GG, Grantcharov TP (2015). The Generic Error Rating Tool: a novel approach to assessment of performance and surgical education in gynecologic laparoscopy. J Surg Educ.

[32] Curry M, Malpani A, Li R, Tantillo T, Jog A, Blanco R, Ha PK, Califano J, Kumar R, Richmon J (2012). Objective assessment in residency-based training for transoral robotic surgery. Laryngoscope.

[33] Gomez ED, Aggarwal R, McMahan W, Bark K, Kuchenbecker KJ (2016). Objective assessment of robotic surgical skill using instrument contact vibrations. Surg Endosc.

[34] Liu M, Purohit S, Mazanetz J, Allen W, Kreaden US, Curet M (2018). Assessment of Robotic Console Skills (ARCS): construct validity of a novel global rating scale for technical skills in robotically assisted surgery. Surg Endosc.

[35] Lovegrove C, Novara G, Mottrie A, Guru KA, Brown M, Challacombe B, Popert R, Raza J, Van der Poel H, Peabody J, Dasgupta P, Ahmed K (2016). Structured and modular training pathway for robot-assisted radical prostatectomy (RARP): validation of the RARP assessment score and learning curve assessment. Eur Urol.

[36] Hussein AA, Hinata N, Dibaj S, May PR, Kozlowski JD, Abol-Enein H, Abaza R, Eun D, Khan MS, Mohler JL, Agarwal P, Pohar K, Sarle R, Boris R, Mane SS, Hutson A, Guru KA (2017). Development, validation and clinical application of Pelvic Lymphadenectomy Assessment and Completion Evaluation: intraoperative assessment of lymph node dissection after robot-assisted radical cystectomy for bladder cancer. BJU Int.

[37] Iqbal U, Jing Z, Ahmed Y, Elsayed AS, Rogers C, Boris R, Porter J, Allaf M, Badani K, Stifelman M, Kaouk J, Terakawa T, Hinata N, Aboumohamed AA, Kauffman E, Li Q, Abaza R, Guru KA, Hussein AA, Eun D (2022). Development and validation of an objective scoring tool for robot-assisted partial nephrectomy: scoring for partial nephrectomy. J Endourol.

[38] Frederick PJ, Szender JB, Hussein AA, Kesterson JP, Shelton JA, Anderson TL, Barnabei VM, Guru K (2017). Surgical competency for robot-assisted hysterectomy: development and validation of a robotic hysterectomy assessment score (RHAS). J Minim Invasive Gynecol.

[39] Goldenberg MG, Lee JY, Kwong JCC, Grantcharov TP, Costello A (2018). Implementing assessments of robot-assisted technical skill in urological education: a systematic review and synthesis of the validity evidence. BJU Int.

[40] Bonrath EM, Zevin B, Dedy NJ, Grantcharov TP (2013). Error rating tool to identify and analyse technical errors and events in laparoscopic surgery. Br J Surg.

[41] Peters JH, Fried GM, Swanstrom LL, Soper NJ, Sillin LF, Schirmer B, Hoffman K, the SAGES FLS Committee (2004). Development and validation of a comprehensive program of education and assessment of the basic fundamentals of laparoscopic surgery. Surgery.

[42] Bann S, Datta V, Khan M, Darzi A (2003). The surgical error examination is a novel method for objective technical knowledge assessment. Am J Surg.

[43] Bonrath EM, Dedy NJ, Zevin B, Grantcharov TP (2013). Defining technical errors in laparoscopic surgery: a systematic review. Surg Endosc.

[44] Curtis NJ, Foster JD, Miskovic D, Brown CSB, Hewett PJ, Abbott S, Hanna GB, Stevenson ARL, Francis NK (2020). Association of surgical skill assessment with clinical outcomes in cancer surgery. JAMA Surg.

[45] Tang B (2020). Observational clinical human reliability analysis (OCHRA) for assessing and improving quality of surgical performance: the current status and future. J Surg Simul.

[46] Coleman M (2009). LAPCO: national training programme for laparoscopic colorectal surgery. Bull R Coll Surg Engl.

[47] Hanna GB, Mackenzie H, Miskovic D, Ni M, Wyles S, Aylin P, Parvaiz A, Cecil T, Gudgeon A, Griffith J, Robinson JM, Selvasekar C, Rockall T, Acheson A, Maxwell-Armstrong C, Jenkins JT, Horgan A, Cunningham C, Lindsey I, Arulampalam T, Motson RW, Francis NK, Kennedy RH, Coleman MG (2022). Laparoscopic colorectal surgery outcomes improved after national training program (LAPCO) for specialists in England. Ann Surg.

