# Supplementary material for: The application of objective clinical human reliability analysis (OCHRA) in the assessment of basic robotic surgical skills
Source: Surg Endosc. 2023 Nov 6;38(1):116–28. doi: 10.1007/s00464-023-10510-2 (PMC10776495; doi:10.1007/s00464-023-10510-2)
Supplement: Supplementary file 1 — Supplementary file1 (DOCX 26 kb) [file 464_2023_10510_MOESM1_ESM.docx]

**Supplementary Table 1 -** Five categorisations for error events provide the OCHRA methodology framework: error type (**A**), consequence of error (**B**), external error mode (**C**), instrument (**D**), and non-error event (**E**).

| **ERROR TYPE *(observed error)*:** | |
| --- | --- |
| **Dissection / Instrument use errors:** | |
| **A.** | Poor visualisation of instrument tip during task e.g. dissection |
| **B.** | Overshoot or undershoot of instrument movement |
| **C.** | Instrument applied with too little distance to structure |
| **D.** | Inappropriate use of diathermy / cutting (tip of instrument visualised) |
| **E.** | Too much/little energy applied with instrument |
| **F.** | Dissection performed in wrong direction |
| **G.** | Diathermy / dissection in wrong tissue plane (plane visible) |
| **H.** | Use of inappropriate instrument to dissect |
| **I.** | Cutting without lifting tissues from underlying structures |
| **J.** | Inappropriate/lack of use of 2^nd/^3^rd^ arm |
| **K.** | Instrument/arm clashing |
| **L.** | Inappropriate/poor use of the camera |
| **M.** | Instrument out of view |
| **N.** | Inappropriate/poor use of endowristed instrument |
| **O.** | Intentional- Inappropriate/unnecessary movement of instrument |
| **P.** | Unintentional- poor/uncontrolled movement of instrument through space |
| **Q.** | Inappropriate/poor suture manipulation e.g. square knot/length of suture |
| **R.** | Excessive force/tension by instrument during task e.g. tightening knot |
|  | |
| **Retraction / Tissue handling errors:** | |
| **S.** | Too much blunt force applied to tissue |
| **T.** | Traction applied with too much tension |
| **U.** | Traction applied with too little tension |
| **V.** | Traction applied in wrong direction |
| **W.** | Inappropriate handling of tissue |
| **X.** | Inappropriate / blunt handling of other structure/object |
| **Y.** | Use of inappropriate instrument to retract |
| **Z.** | Avulsion of tissue |

**A** – Error types for generic robotic surgical skills. Further subcategorised into Dissection/Instrument use errors and Retraction/Tissue handling errors.

| **CONSEQUENCE:** | |
| --- | --- |
| **1.** | Suture fray |
| **2.** | Suture break |
| **3.** | Object (needle/ring) dropped |
| **4.** | Object flies out of view |
| **5.** | Needle bend/at risk of |
| **6.** | Needle snapped off suture |
| **7.** | Needle snapped |
| **8.** | Tissue/foam pad damaged |
| **9.** | Poor apposition of tissue |
| **10.** | Unequal suture distance |
| **11.** | Partial thickness or poor stitch placement |
| **12.** | Back or side wall caught |
| **13.** | Risk of injury to other structure/minor collision |
| **14.** | Injury to other structure |
| **15.** | Delay in progress of procedure |
| **16.** | Inadequate dissection |
| **17.** | Bleeding (ooze) |
| **18.** | Bleeding (significant/pulsatile) |
| **19.** | Instrument clash |

**B** - Consequence of errors for general robotic surgical skills. Consequence severity deemed not feasible within basic robotic skills, as errors are usually binary with no true severity rate, therefore excluded from our analysis.

| **EXTERNAL ERROR MODE:** | |
| --- | --- |
| **a.** | Step is not done |
| **b.** | Step is partially completed |
| **c.** | Step is repeated |
| **d.** | Second step is done in addition |
| **e.** | Second step is done instead of first step |
| **f.** | Step is done out of sequence |
| **g.** | Step is done with too much force / speed / depth / distance / time / rotation |
| **h.** | Step is done with too little force / speed / depth / distance / time / rotation |
| **i.** | Step is done in wrong orientation/direction/point in space |
| **j.** | Step is done on/with the wrong object |
| Procedural errors = EEMs a–f.  Executional errors = EEMs g–j. | |

**C** -External error modes of errors for general robotic surgical skills. Procedural errors (a-f). Executional errors (g-j).

| **INSTRUMENT:** | |
| --- | --- |
| **1.** | Hook Diathermy |
| **2.** | Finger-switch diathermy |
| **3.** | Diathermy forceps |
| **4.** | Harmonic Scalpel / Ligasure |
| **5.** | Blunt grasper (e.g. Johan) |
| **6.** | Fine grasper (Marylands, needle driver) |
| **7.** | Swab |
| **8.** | Suction |
| **9.** | Scissors |
| **10.** | Forceps |
| **11.** | Stapler |
| **12.** | Bowel clamp |
| **13.** | Liga-clip applicator |
| **14.** | Finger – blunt dissection |
| **15.** | Retractor |
| **16.** | Camera endoscope |

**D -** Instruments used when error occurs.

| **NON-ERROR EVENT:** | |
| --- | --- |
| **Preparatory Step:** | |
| a. | Adjust hold or camera view to improve visualisation |
| b. | Adjust hold to improve orientation |
| c. | Adjust hold to improve traction |
| d. | Adjust hold to separate structures |
| e. | Search for structure to dissect/divide or avoid/preserve |
| **Recovery mechanisms:** | |
| f. | Continue uninterrupted |
| g. | Perform step previously omitted |
| h. | Requires repetition of step |
| j. | Corrective action within subtask |
| k. | Change in subtask/sequence |
| **Other events:** | |
| l. | Cleaning of camera |
| m. | Inadequate camera view |
| n. | Port dislocation |
| o. | Port adjustment |
| p. | Additional action/event |

**E -** Non-error event is the event that follows or should follow an error event.
