# Supplementary material for: The application of objective clinical human reliability analysis (OCHRA) in the assessment of basic robotic surgical skills
Source: Surg Endosc. 2023 Nov 6;38(1):116–28. doi: 10.1007/s00464-023-10510-2 (PMC10776495; doi:10.1007/s00464-023-10510-2)
Supplement: Supplementary file 2 — Supplementary file2 (DOCX 31 kb) [file 464_2023_10510_MOESM2_ESM.docx]

**Supplementary Table 2 -** Hierarchical task analysis for sea spikes (**A**), ring rollercoaster (**B**), big dipper needle driving (**C**) and knot tying (**D**) dry lab and VR tasks. Correct actions for performing each subtask are described within the aim.

| **Sea Spikes** | | | |
| --- | --- | --- | --- |
| **Task Zone** | **Subtask** | **Start Point** | **Aim** |
| 1. Picking up ring | 1. Visualisation of instruments | Rings, cones, or instruments appears on screen  *(Or completion of step 4 [Movement of instruments back to starting position])* | Both instruments within field of vision by moving camera appropriately |
|  | 1. Visualisation of ring |  | Ring within field of vision by moving camera appropriately |
|  | 1. Grasping of ring |  | Appropriate angle of instrument and force used to pick up ring |
| 1. Movement of ring in space | 1. Visualisation of ring and instruments | Instrument grasping ring | Instrument grasping ring remains within field of vision by moving camera appropriately |
|  | 1. Efficient movement/appropriate path of instrument & ring |  | Move ring efficiently to appropriate cone whilst avoiding contact with other structures |
| 1. Placement of ring on cone | 1. Visualisation of instrument, ring, and cone | Instrument with ring grasped above appropriate cone | Instrument grasping ring and cone within field of vision by moving camera appropriately |
|  | 1. Placement of ring on cone |  | Appropriate angle of ring and force applied to place ring on cone. Avoiding instrument-cone contact |
| 1. Movement of instruments back to starting position | 1. Visualisation of instruments | Ring placed on cone  *(ring turns green on VR)* | Both instruments remain within field of vision by moving camera appropriately |
|  | 1. Efficient movement/appropriate path of instrument & ring |  | Move instruments/camera efficiently to starting position whilst avoiding contact with other structures |

**A –** Task analysis for Sea spikes dry lab and VR tasks.

| **Ring Rollercoaster** | | | |
| --- | --- | --- | --- |
| **Task Zone** | **Subtask** | **Start Point** | **Aim** |
| 1. Picking up ring | 1. Visualisation of instruments | Rings, cones, or instruments appears on screen  *(Or completion of step 4 [Movement of instruments back to starting position])* | Both instruments within field of vision by moving camera appropriately |
|  | 1. Visualisation of ring |  | Ring within field of vision by moving camera appropriately |
|  | 1. Grasping of ring |  | Appropriate angle of instrument and force used to pick up ring |
| 1. Movement of ring along the rollercoaster | 1. Visualisation of ring and instruments | Instrument grasping ring | Instrument grasping ring remains within field of vision by moving camera appropriately |
|  | 1. Efficient movement/appropriate path of instrument & ring along straight sections of rollercoaster |  | Move ring efficiently along straight sections of rollercoaster avoiding contact |
|  | 1. Efficient movement of ring along curved sections of rollercoaster |  | Move ring efficiently along curved sections of rollercoaster avoiding contact. Appropriate pronation/supination |
| 1. Transfer of ring from instrument to instrument   *(After completion of task zone to continue with task zone 2: Movement of ring along rollercoaster)* | 1. Visualisation of ring and instruments | Varies depending on candidate choice | Both instruments and ring within field of vision |
|  | 1. Both instruments grasping ring |  | Appropriate angle of instruments and force used to grasp ring |
|  | 1. Release of instrument grasping ring |  | Successful transfer of ring to the other instrument |
| 1. Movement of instruments back to starting position | 1. Visualisation of instruments | Ring placed at end of rollercoaster *(ring turns green on VR)* | Both instruments remain within field of vision by moving camera appropriately |
|  | 1. Efficient movement/appropriate path of instrument & ring |  | Move instruments efficiently to starting position whilst avoiding contact with other structures |

**B –** Task Analysis for Ring rollercoaster dry lab and VR tasks.

| **Big Dipper Needle Driving** | | | |
| --- | --- | --- | --- |
| **Task Zone** | **Subtask** | **Start Point** | **Aim** |
| 1. Preparing needle for tissue insertion | 1. Visualisation of instruments | Needle & instruments appears on screen  *(Or completion of step 4 [Exit of needle from tissue])* | Both instruments within field of vision by moving camera appropriately |
|  | 1. Visualisation of needle |  | Needle within field of vision by moving camera appropriately |
|  | 1. Picking up needle |  | Appropriate angle of instrument and force used to grasp and pick up needle |
|  | 1. Transfer of needle from one instrument to the other   *(Optional step – can be in addition to or replace step 1.3 [picking up needle] if appropriate)* |  | Efficient use of both instruments simultaneously to transfer the needle proficiently |
|  | 1. Needle grasped at 1/2 to 2/3 from needle tip |  | Appropriate spatial awareness and instrument control to grasp needle at correct location |
|  | 1. Correct orientation of needle |  | Needle perpendicular to instrument |
| 1. Insertion of needle into tissue | 1. Visualisation of instruments | Instrument grasping needle once orientated appropriately | Both instruments within field of vision by moving camera appropriately |
|  | 1. Visualisation of needle |  | Needle within field of vision by moving camera appropriately |
|  | 1. Correct point of entry of needle |  | Needle inserted into outlined mark on tissue |
|  | 1. Needle inserted at 90° to tissue |  | Appropriate motion control to insert needle at 90° to tissue |
| 1. Driving of needle through tissue | 1. Visualisation of instruments | Needle inserted into tissue | Both instruments within field of vision by moving camera appropriately |
|  | 1. Visualisation of needle |  | Needle within field of vision by moving camera appropriately |
|  | 1. Appropriate path of needle through tissue |  | Smooth and controlled movement of needle though tissue |
|  | 1. Appropriate depth of needle through tissue |  | Needle not too superficial or too deep when driving needle through the tissue |
| 1. Exit of needle from tissue | 1. Visualisation of instruments | Needle tip exiting tissue | Both instruments within field of vision by moving camera appropriately |
|  | 1. Visualisation of needle |  | Needle within field of vision by moving camera appropriately |
|  | 1. Correct point of exit of needle |  | Appropriate bite distance for exit of needle |
|  | 1. Needle pulled out along its body/curve |  | The needle is not grasped by the needle tip |

**C –** Task Analysis for Big dipper needle driving dry lab and VR tasks.

| **Knot Tying** | | | |
| --- | --- | --- | --- |
| **Task Zone** | **Subtask** | **Start Point** | **Aim** |
| 1. Preparing suture thread for making loops | 1. Visualisation of instruments | Suture thread & instruments appears on screen  *(Or completion of step 4 [tightening of knot])* | Both instruments within field of vision by moving camera appropriately |
|  | 1. Visualisation of suture thread |  | Thread within field of vision by moving camera appropriately |
|  | 1. Picking up suture thread long end |  | Appropriate angle of instrument and force used to grasp and pick up thread. Appropriate C-loop or reverse C-loop formed. |
|  | 1. Transfer suture thread from one instrument to the other   *(Optional step – can be in addition or replace step 1.3 [picking up suture thread long end] if appropriate)* |  | Efficient use of both instruments simultaneously to transfer the needle proficiently |
| 1. Wrapping suture thread around the instrument | 1. Visualisation of instruments | Instrument grasping long end of thread with appropriate instrument | Both instruments within field of vision by moving camera appropriately |
|  | 1. Visualisation of suture thread |  | Thread within field of vision by moving camera appropriately |
|  | 1. Wrapping suture thread long end around instrument (twice for the first throw and once for the subsequent two throws) |  | Controlled movements of both instruments to wrap thread around the instrument. No instrument clashes |
| 1. Pulling suture thread short tail through loop | 1. Visualisation of instruments | Thread wrapped around instrument | Both instruments within field of vision by moving camera appropriately |
|  | 1. Visualisation of suture thread |  | Thread within field of vision by moving camera appropriately |
|  | 1. Grasping of short tail with appropriate instrument |  | Appropriate angle of instrument and force to grasp thread short tail |
|  | 1. Pulling short tail through loop |  | Efficient movement of instrument to pull short tail through loop |
| 1. Tightening knot | 1. Visualisation of instruments | Short tail pulled through loop | Both instruments within field of vision by moving camera appropriately |
|  | 1. Visualisation of suture thread |  | Thread within field of vision by moving camera appropriate |
|  | 1. Pull both ends of thread in opposite directions |  | Appropriate tension and control to provide to tighten knot |

**D -** Task analysis for Knot tying dry lab and VR tasks.
